# Supplementary material for: Evaluation of Growth, Yield, and Biochemical Attributes of Bitter Gourd (Momordica charantia L.) Cultivars under Karaj Conditions in Iran
Source: Plants (Basel). 2021 Jul 5;10(7):1370. doi: 10.3390/plants10071370 (PMC8309235; doi:10.3390/plants10071370)
Supplement: Supplementary file 1 [file plants-10-01370-s001.zip › plants-1254040-supplementary.pdf]

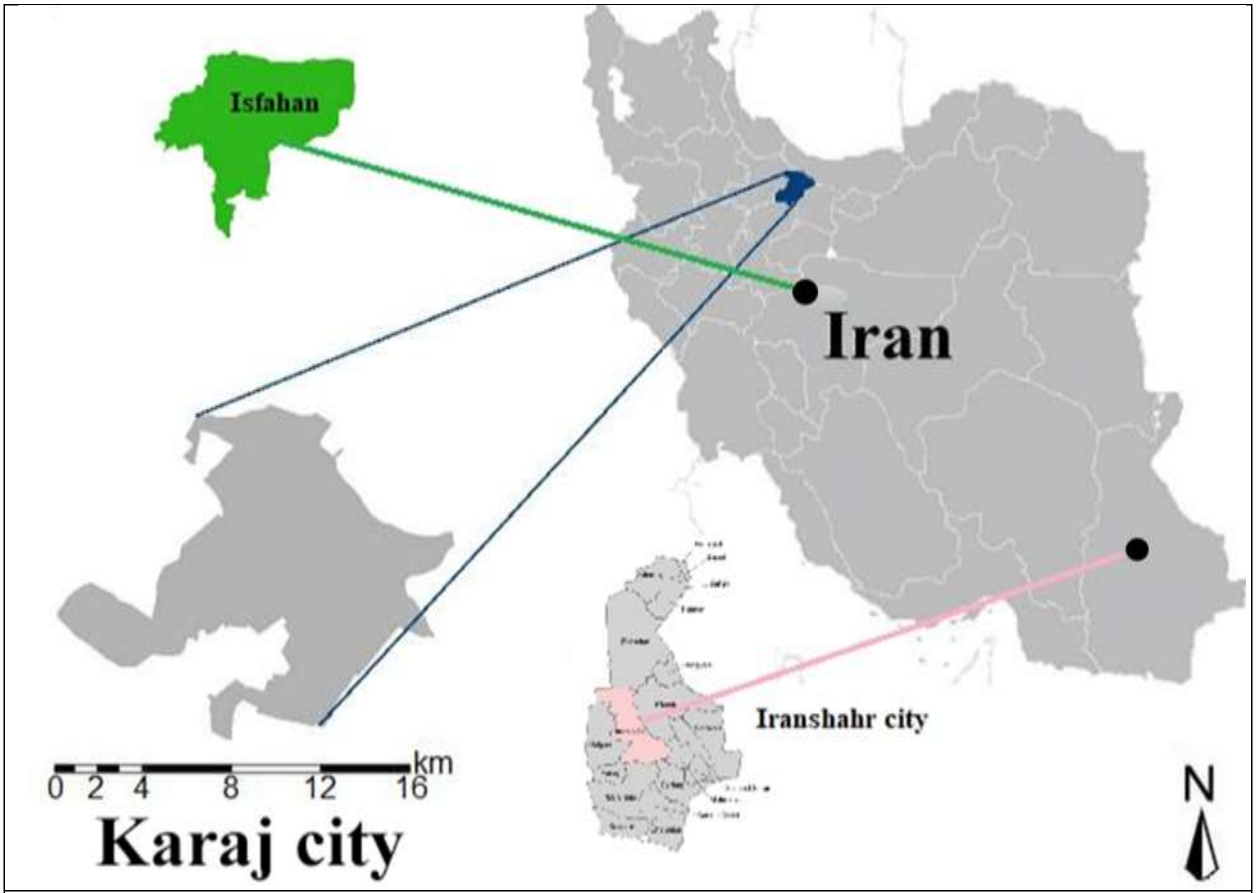

Figure S1. Geographical map of the investigation cite (Karaj) and other Iranian Cite that the seeds were harvested.

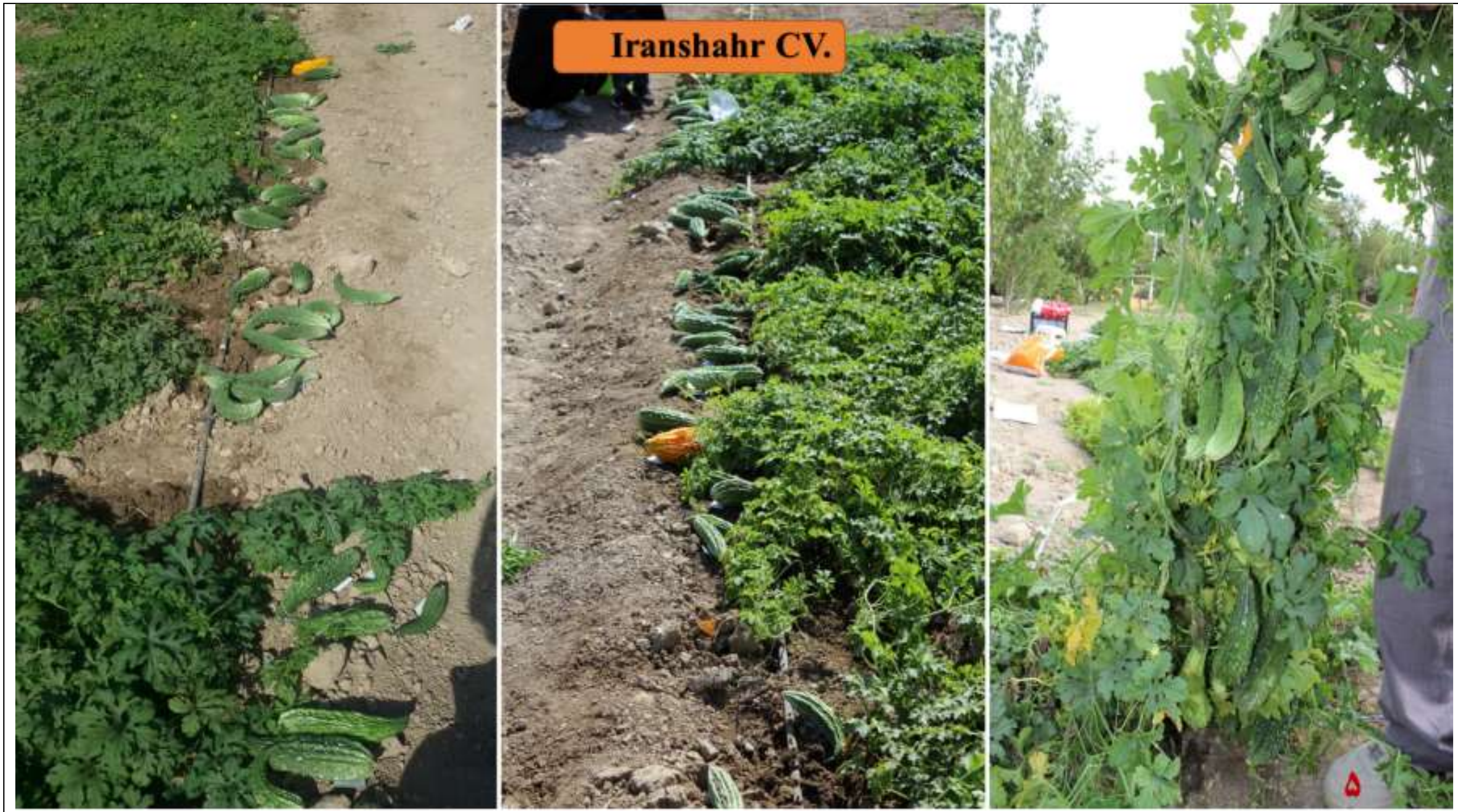
Iranshahr CV.
۵

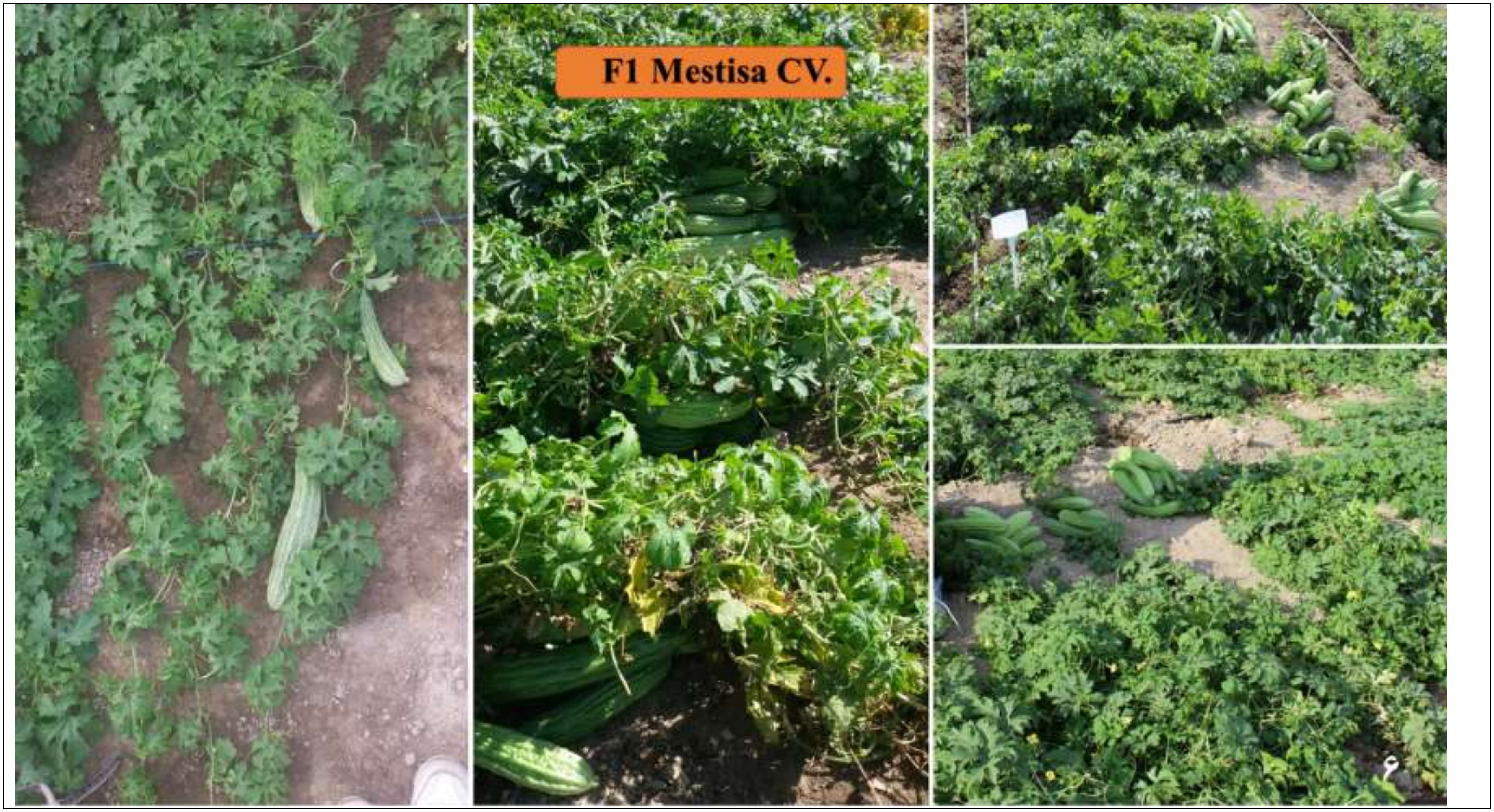
F1 Mestisa CV.

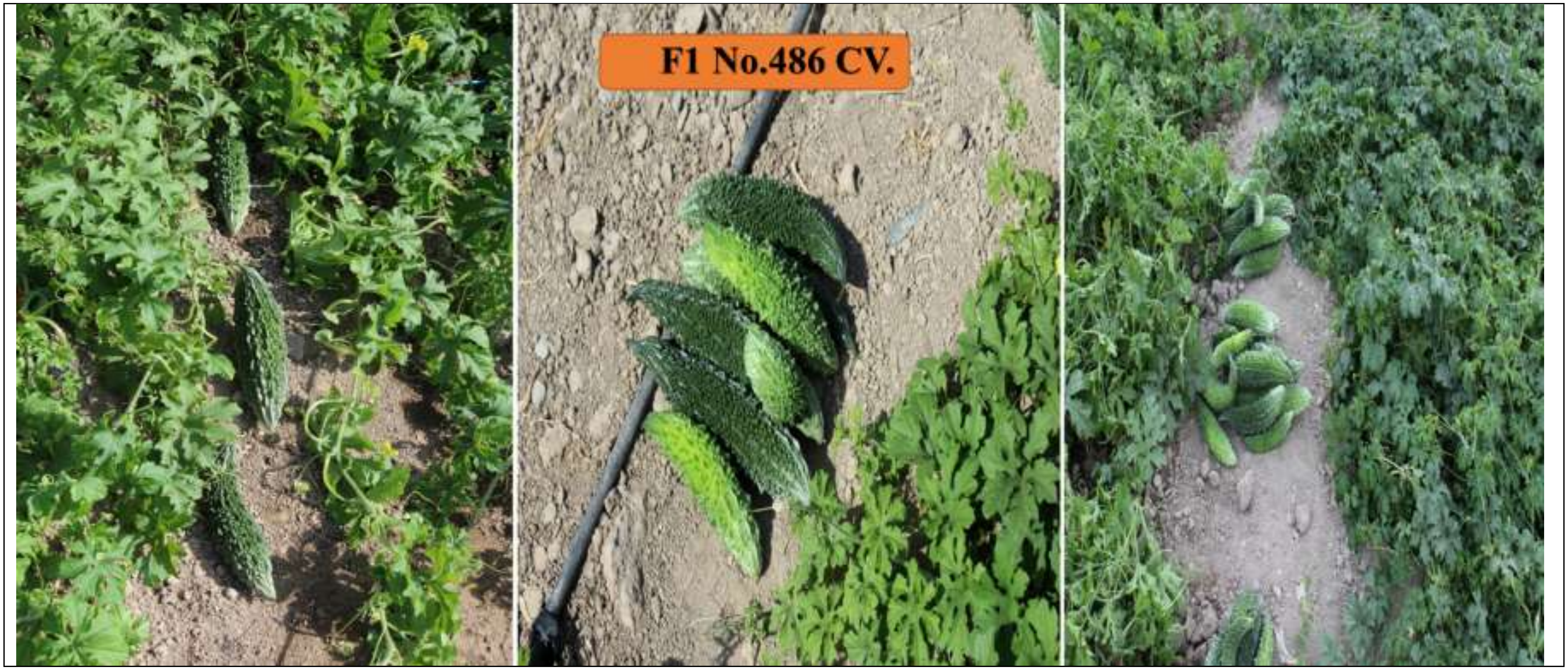
F1 No.486 CV.

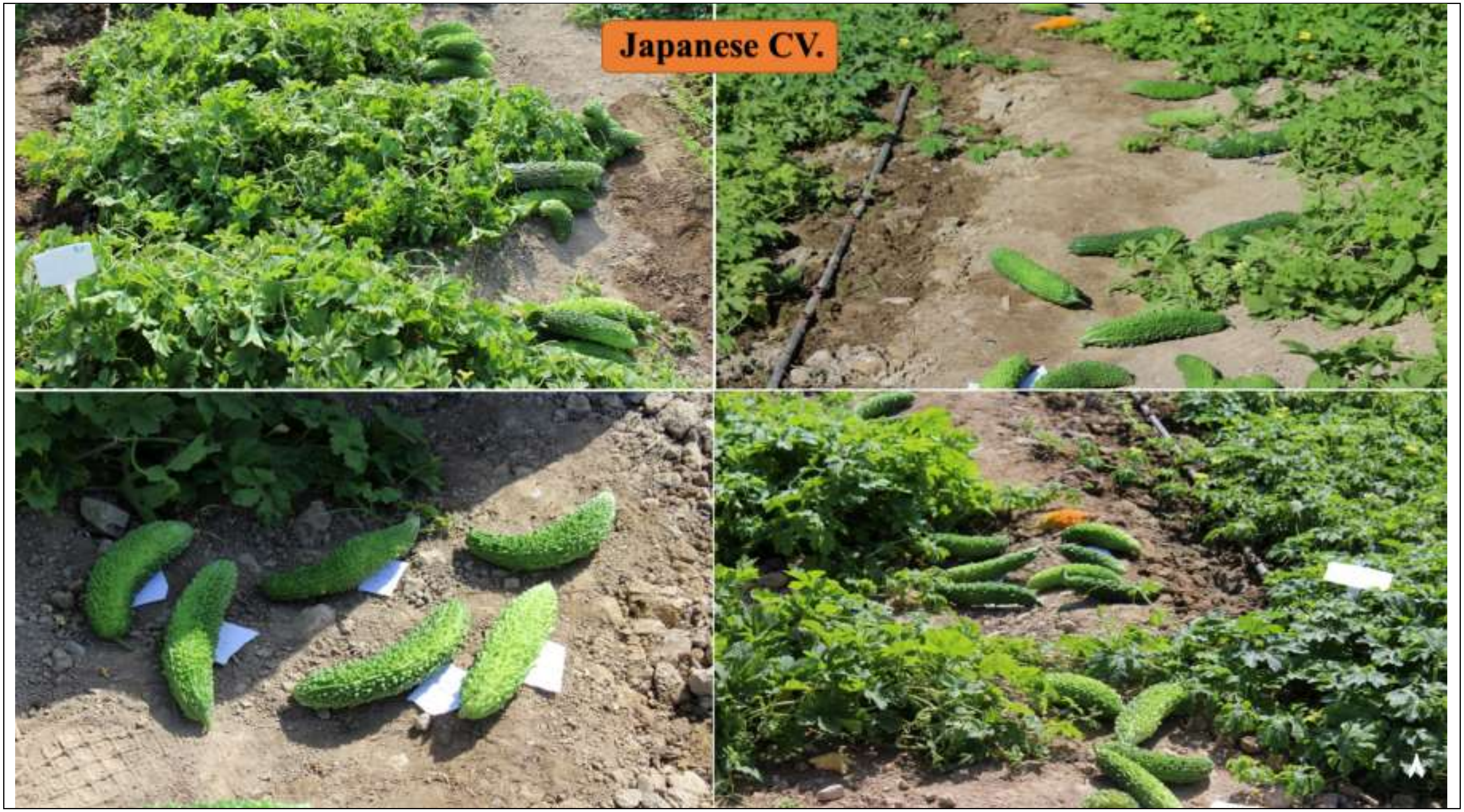
Japanese CV.

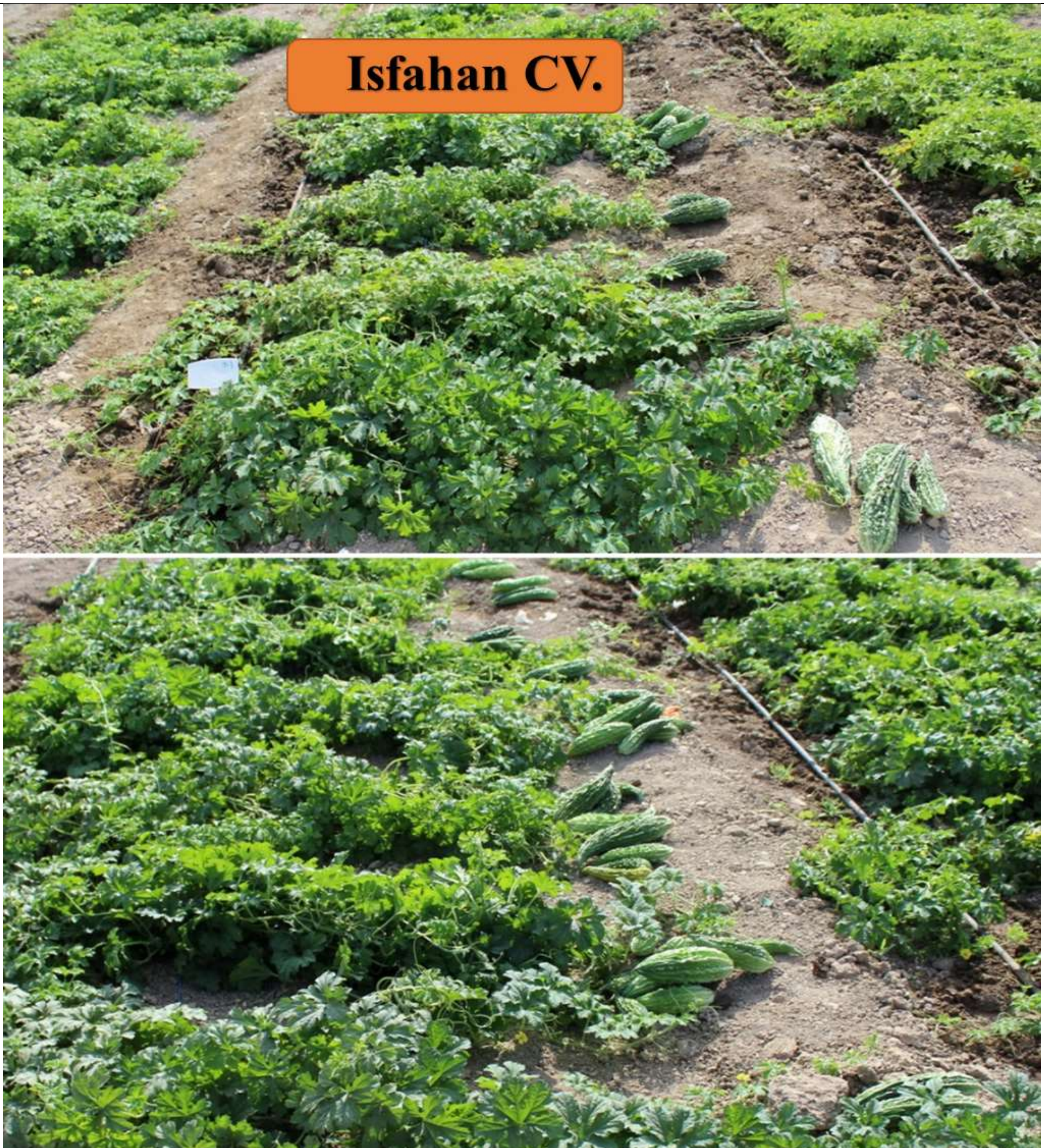
Isfahan CV.

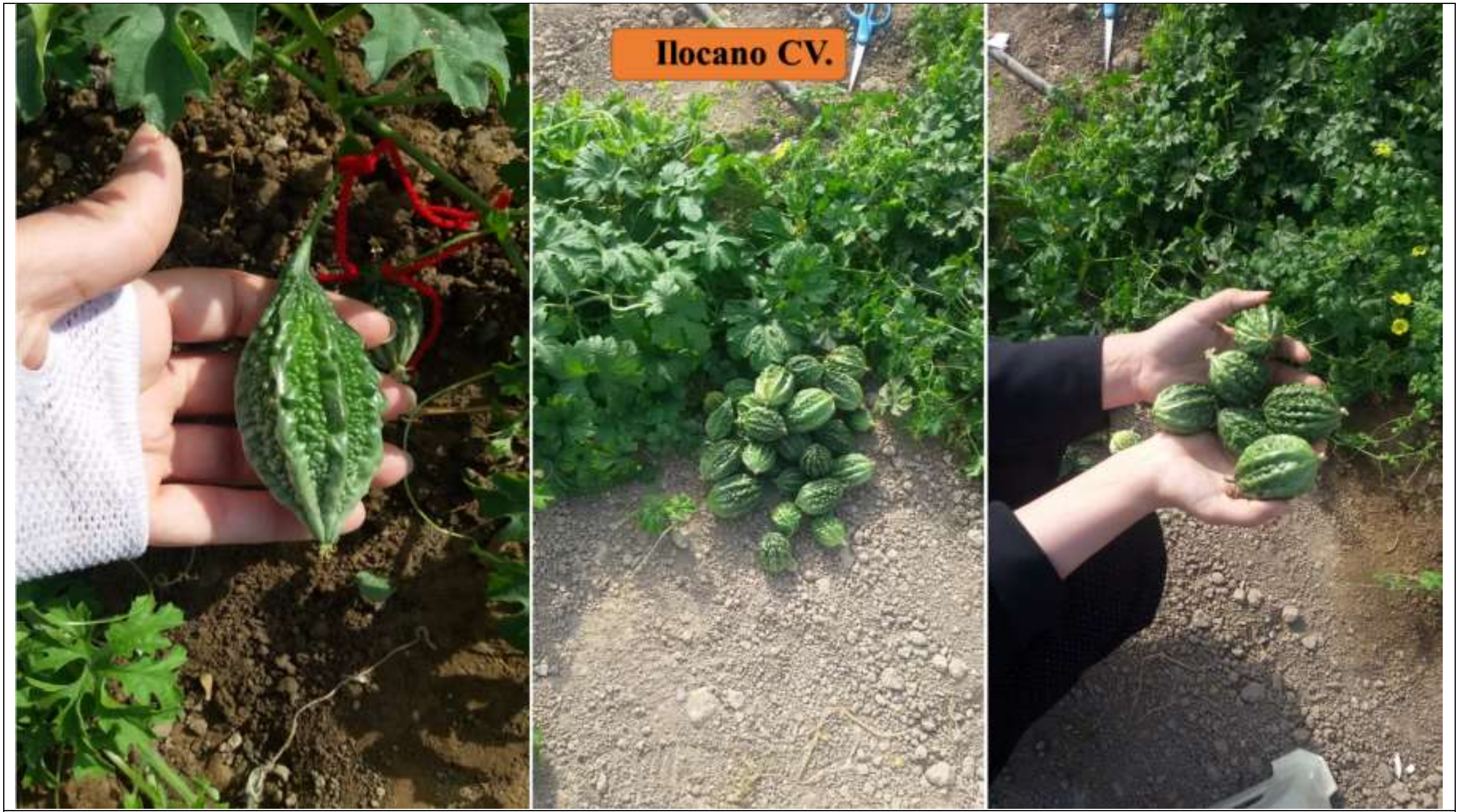

Figure S2. Bitter gourd cultivars studied in the research
